# Factors Associated with Acute Lower Respiratory Infections in Children Under Five at a Peruvian Emergency Hospital

**DOI:** 10.3390/children13081120

**Published:** 2026-08-21

**Authors:** Anthony D. Mautino-Reyes, Fiorella F. Fuentes-Cosme, Miguel A. Arce-Huamani

**Affiliations:** 1 Programa Academico de Medicina Humana, Facultad de Ciencias de la Salud, Universidad Privada San Juan Bautista, Lima 15046, Peru; anthoni.mautino@upsjb.edu.pe; 2 Programa Academico de Medicina Humana, Facultad de Ciencias de la Salud, Universidad Privada Norbert Wiener, Lima 15046, Peru; a2021201256@uwiener.edu.pe

**Keywords:** acute lower respiratory infection, pneumonia, bronchiolitis, children under five, breastfeeding, immunization, passive smoking

## Abstract

**Highlights:**

**What are the main findings?**

- Acute lower respiratory infection (ALRI), defined as a physician-recorded diagnosis of pneumonia or bronchiolitis, accounted for 47.8% of ARI diagnoses in the analytical sample of children under five treated at a Peruvian emergency hospital.
- Age 0 to <2 years, absence of exclusive breastfeeding, birth weight < 1000 g, incomplete immunizations, and passive smoking exposure were independently associated with a higher prevalence of ALRI.

**What is the implication of the main finding?**

- In pediatric emergency settings, these factors may help identify children more likely to present with ALRI.
- Strengthening exclusive breastfeeding, timely childhood immunization, follow-up of extremely low-birth-weight children, and smoke-free home environments may help reduce the burden of ALRI in similar emergency-care settings.

**Abstract:**

**Background/Objectives**: Acute lower respiratory infections (ALRIs) are an important cause of morbidity among children under five years of age. This study aimed to identify factors associated with ALRI among children under five treated at a Peruvian emergency hospital. **Methods**: We conducted an observational, analytical, retrospective cross-sectional study using medical records of children treated in the pediatric emergency department of Hospital de Emergencias José Casimiro Ulloa, Lima, Peru, between January and December 2024. ALRI was defined as a physician-recorded diagnosis of pneumonia or bronchiolitis; the comparison group comprised children with acute bronchitis or upper respiratory tract infection. Crude and adjusted prevalence ratios (PRs) with 95% confidence intervals (CIs) were estimated using Poisson regression with robust variance. **Results**: The final sample included 322 children, of whom 154 (47.8%) had ALRI. In the adjusted model, age 0 to <2 years (aPR = 1.27; 95% CI: 1.03–1.56), absence of exclusive breastfeeding (aPR = 1.24; 95% CI: 1.02–1.51), birth weight < 1000 g (aPR = 1.43; 95% CI: 1.09–1.87), incomplete immunizations (aPR = 1.28; 95% CI: 1.07–1.53), and passive smoking exposure (aPR = 1.38; 95% CI: 1.15–1.65) were independently associated with higher ALRI prevalence. **Conclusions**: ALRI accounted for nearly half of the ARI diagnoses in this pediatric emergency analytical sample. Younger age, absence of exclusive breastfeeding, extremely low birth weight, incomplete immunizations, and passive smoking exposure were independently associated with ALRI and may help identify children requiring closer clinical assessment and preventive interventions.

## 1. Introduction

Acute lower respiratory infections (ALRIs) remain a major cause of pediatric morbidity, hospitalization, and preventable mortality, particularly among children under five years of age. In 2019, approximately 5.30 million deaths occurred among children younger than five worldwide, with lower respiratory infections remaining among the leading infectious causes of death in this age group [1]. Respiratory syncytial virus (RSV) alone was estimated to cause 33.0 million ALRI episodes, 3.6 million hospital admissions, 26,300 in-hospital deaths, and 101,400 overall deaths among children aged 0–60 months in 2019 [2]. This burden is disproportionately concentrated in low- and middle-income countries, where more than 95% of RSV-associated ALRI episodes and most RSV-attributable deaths occur [3]. Hospital-based surveillance using the severe acute respiratory infection (SARI) case definition captures a clinically more severe spectrum of respiratory disease; in a tertiary-care study in India, respiratory viruses were detected in 69% of pediatric SARI cases, with RSV identified in 31% [4].

The etiology of respiratory infections in young children is diverse and varies according to age, season, comorbidities, vaccination status, and diagnostic capacity. In Peru, hospitalized patients with RSV and influenza have shown distinct clinical and epidemiological characteristics, supporting the need for locally generated evidence to guide pediatric respiratory care [5]. Previous Peruvian molecular surveillance detected respiratory viruses in 33.5% of children with acute respiratory infections (ARIs), with RSV-A being the most frequently identified agent at 15.3% [6]. Other Peruvian hospital data suggest that bacterial pathogens such as Bordetella pertussis may also be underrecognized among children hospitalized with ARI [7]. Multicenter SARI surveillance has further shown that RSV and rhinovirus may be associated with greater clinical severity than influenza and adenovirus, including pneumonia, intensive care admission, and fatal outcomes [8].

Vaccination coverage also remains relevant in the Peruvian context. According to the 2024 Demographic and Family Health Survey (ENDES), 73.9% of children younger than 36 months in the Department of Lima had received all vaccines appropriate for their age. Coverage was higher for individual vaccines, reaching 99.2% for BCG, 90.8% for measles-containing vaccine, 91.9% for the third dose of pentavalent vaccine, and 93.1% for the third dose of poliovirus vaccine. The age-appropriate complete schedule assessed by ENDES also included three doses of pneumococcal vaccine [9]. These data indicate that gaps in completion of the childhood immunization schedule persist even in Lima, providing relevant context for evaluating immunization status in relation to pediatric respiratory infections.

Several biological, household, and environmental factors have been associated with ALRI and other clinically important respiratory infections in children under five. An updated systematic review identified prematurity, low birth weight, passive smoking, daycare attendance, chronic disease, congenital heart disease, and previous asthma as risk factors for RSV-associated ALRI [10]. Indoor air pollution is also relevant, particularly in low- and middle-income countries, where exposure to biomass fuels, traditional stoves, secondhand smoke, poor ventilation, and stunting has been associated with ARI [11]. A hospital-based matched case–control study in Bangladesh found that household overcrowding, unclean cooking fuels, in-house smoking, unimproved sanitation, higher birth order, and preterm birth were associated with childhood ARI, whereas exclusive breastfeeding was protective [12]. In Uganda, caregiver-reported ARI symptoms affected 40.3% of children under five, with higher risk among younger children and those born to teenage mothers [13].

In Peru, available evidence on childhood respiratory infections has largely focused on population-based ARI symptoms, pathogen distribution, or clinical and epidemiological characteristics in hospitalized children [5,6,7,14]. Evidence evaluating adjusted associations with physician-diagnosed ALRI in pediatric emergency populations remains limited. Hospital de Emergencias José Casimiro Ulloa is a public emergency hospital in Lima, where pediatric respiratory conditions constitute an important component of emergency presentations [15].

Accordingly, the contribution of this study lies not in identifying previously unknown risk factors, but in providing context-specific adjusted estimates of their associations with ALRI in an understudied Peruvian emergency-care population. By simultaneously evaluating perinatal characteristics, breastfeeding, immunization status, household conditions, and passive smoking exposure, the study provides locally relevant evidence that may help prioritize risk assessment and preventive counseling in comparable emergency settings. Therefore, the primary objective of this study was to estimate the crude and adjusted associations between host, perinatal, sociodemographic, and environmental factors and ALRI among children under five treated at a Peruvian emergency hospital. The secondary objectives were to describe the frequency of ALRI in the analytical sample and to compare the distribution of the evaluated characteristics between children with ALRI and those with other acute respiratory infections.

## 2. Materials and Methods

### 2.1. Study Design and Setting

We conducted a quantitative, observational, analytical, retrospective cross-sectional study among children under five years of age in Lima, Peru. The study was carried out at Hospital de Emergencias José Casimiro Ulloa, a public hospital of the Peruvian Ministry of Health dedicated to emergency and urgent care in Lima, Peru. The hospital provides continuous 24-h emergency services and receives a substantial volume of emergency presentations, including pediatric respiratory conditions [15]. Data were obtained from medical records of children attended in the pediatric emergency department between January and December 2024.

The primary objective was to estimate the association between the evaluated host, perinatal, sociodemographic, and environmental factors and ALRI. Secondary objectives were to describe the frequency of ALRI in the analytical sample and to compare the distribution of these characteristics between children with ALRI and those with other acute respiratory infections. Because the study was based on retrospective review of routinely collected clinical records, no intervention was performed and no follow-up of participants was required.

### 2.2. Study Population, Sample Size, and Sampling

The source population consisted of children younger than five years with a recorded diagnosis of acute respiratory infection (ARI) who were attended in the pediatric emergency department of Hospital de Emergencias José Casimiro Ulloa between January and December 2024. According to the institutional registry, 2484 children met these initial registry-based criteria during the study period.

Because this was a retrospective cross-sectional study based on a hospital registry, the sample size was estimated for a single proportion with finite population correction. This calculation was originally used to determine the minimum number of medical records required to estimate the expected frequency of acute respiratory infection in the finite source population. The following formula was applied:

*n* = [*N* × *Z*^2^ × *p* × *q*]/[*d*^2^ × (*N* − 1) + *Z*^2^ × *p* × *q*]

where *N* was the finite source population, *Z* was the standard normal value for a 95% confidence level, *p* was the expected proportion, *q* was calculated as 1 − *p*, and *d* was the absolute precision. The expected proportion was based on a previous hospital-based descriptive cross-sectional study among children under five years of age, which reported an acute respiratory infection prevalence of 36.67% (95% CI: 32.99–40.34%) [16]. To obtain a conservative estimate, the upper limit of this confidence interval was used as the expected proportion. Therefore, the parameters used were *N* = 2484, *Z* = 1.96, *p* = 0.4034, *q* = 0.5966, and *d* = 0.05. This calculation yielded a minimum required sample size of 322 children.

Because the primary objective of the study was analytical, we additionally assessed whether the calculated sample was adequate to evaluate associations between explanatory factors and ALRI. For this complementary assessment, we used incomplete age-appropriate immunization as a clinically relevant exposure because comparable data were available from a recent hospital-based study of children under five by Islam et al. [12]. In that study, incomplete immunization was present in approximately 20.7% of children with ARI and 7.5% of controls. Assuming a two-sided significance level of 0.05, 80% power, and a 1:1 allocation ratio, comparison of these proportions yielded a minimum required sample size of approximately 206 participants. Therefore, the final analytical sample of 322 children exceeded this complementary analytical sample-size requirement. Because the external study did not use the same ALRI-versus-other-ARI comparison as the present analysis, this calculation was considered a complementary benchmark of sample adequacy rather than a direct outcome-specific sample-size determination.

The sampling frame comprised the 2484 medical records of children younger than five years with a registered diagnosis of ARI during the study period. A simple random sample without replacement was drawn from this sampling frame, such that each medical record had an equal probability of selection and previously selected records could not be selected again. Initially, 322 medical records were randomly selected and subsequently assessed against the predefined eligibility criteria. Of these, 25 were excluded: 12 because of incomplete essential information, seven because detailed medical record review showed that the diagnosis did not meet the study definition of ARI, four because the record did not correspond to the 2024 study period, and two for other documented exclusion reasons. Each excluded record was replaced by a new random draw from the remaining unsampled records using the same simple random sampling procedure. All 25 replacement records met the eligibility criteria. Therefore, 347 medical records were reviewed during the complete selection and replacement process, yielding a final analytical sample of 322 eligible children.

### 2.3. Eligibility Criteria

Children were eligible if they were younger than five years of age, had been attended in the pediatric emergency department of Hospital de Emergencias José Casimiro Ulloa between January and December 2024, and had a confirmed diagnosis of acute respiratory infection recorded in the medical record.

Medical records were excluded when essential information was incomplete or unavailable, including age, sex, reason for consultation, final diagnosis, or the main variables required for the analysis. Records that did not meet the operational definition of acute respiratory infection or did not correspond to the study period were also excluded from the final dataset.

When an initially selected record was excluded, a replacement record was randomly drawn without replacement from the remaining sampling frame until the required analytical sample of 322 eligible records was reached.

Pediatric comorbidities were not used as exclusion criteria; therefore, the presence of chronic underlying conditions did not determine eligibility for inclusion in the analytical sample.

### 2.4. Variables and Definitions

The primary outcome was acute lower respiratory infection (ALRI). ALRI was defined as a physician-recorded final diagnosis of pneumonia or bronchiolitis, consistent with definitions used in published pediatric ALRI burden studies [2,17]. The comparison group comprised children with other acute respiratory infection diagnoses, including acute bronchitis or upper respiratory tract infection. Classification was based on the final diagnosis documented in the medical record during the emergency-department evaluation. For pneumonia, the study relied on the physician-recorded final diagnosis and did not independently adjudicate the diagnosis. Information on the diagnostic work-up, including whether chest radiography or lung ultrasound was performed, was not systematically collected; therefore, the imaging modality used in individual pneumonia cases could not be determined retrospectively. This classification was not intended to represent the World Health Organization surveillance case definition of severe acute respiratory infection (SARI).

Throughout the manuscript, ARI is used as the broader term encompassing all acute respiratory infection diagnoses included in the study, whereas ALRI refers specifically to the study outcome of pneumonia or bronchiolitis. The abbreviation SARI is reserved exclusively for external studies or surveillance systems that explicitly applied a severe acute respiratory infection case definition.

The explanatory variables were grouped into host and perinatal factors, sociodemographic factors, and environmental factors. Host and perinatal factors included child age, sex, exclusive breastfeeding, birth-weight category, gestational age at birth, and complete immunization status. Child age was categorized as 0 to <2 years and 2 to <5 years. Birth weight was categorized as 1500 to <2500 g, 1000 to <1500 g, and <1000 g. Gestational age was categorized as term birth, defined as ≥37 weeks, and preterm birth, defined as <37 weeks. Exclusive breastfeeding and complete immunizations were categorized as yes or no according to the information recorded in the medical record.

Sociodemographic factors included household overcrowding, housing material, maternal age, and maternal occupation. Household overcrowding was defined as more than three persons residing per room in the dwelling, excluding the kitchen, bathroom, hallways, and garage, according to the criterion established by the Peruvian National Institute of Statistics and Informatics (INEI) [18]. It was categorized as present or absent. Housing material was classified as masonry or permanent material versus prefabricated material. Maternal age was categorized as 15 to <30 years, 30 to <45 years, and 45 to <55 years. Maternal occupation was categorized as homemaker, part-time work, and full-time work.

Environmental factors included passive smoking exposure, defined as documented postnatal household tobacco smoke exposure and categorized as yes or no according to information recorded in the medical record. District of origin was also collected in the data extraction form when available.

### 2.5. Data Collection Procedures

After obtaining institutional and ethics approvals, the research team coordinated with the Statistics and Informatics Department and the pediatric emergency department of Hospital de Emergencias José Casimiro Ulloa to access the eligible medical records.

Data were collected through retrospective review of clinical records using a structured data extraction form developed for the study. The form was reviewed through expert judgment and included four sections. The first section recorded the type of acute respiratory infection, including acute bronchitis, bronchiolitis, pneumonia, and upper respiratory tract infection. The second section collected host and perinatal factors, including child age, sex, exclusive breastfeeding, birth weight, gestational age at birth, and immunization status. The third section collected sociodemographic factors, including household overcrowding, housing material, maternal age, and maternal occupation. The fourth section collected environmental factors, including passive smoking exposure and district of origin.

The extracted information was entered into a Microsoft Excel 2024 database. The database was coded, reviewed, and cleaned before statistical analysis. Consistency checks were performed to identify missing, duplicated, or implausible values. Records with incomplete information for the main outcome or essential explanatory variables were excluded from the final analytical dataset.

### 2.6. Statistical Analysis

Categorical variables were summarized using absolute frequencies and percentages. Percentages were calculated using the number of non-missing observations available for each variable. Missing values were minimal for the study variables, and no imputation procedures were performed.

Bivariate analyses were performed to compare the distribution of explanatory variables between children with ALRI and those in the comparison group. Pearson’s chi-square test or Fisher’s exact test was used, as appropriate, according to expected cell frequencies. A two-sided *p* value < 0.05 was considered statistically significant in bivariate comparisons.

To estimate associations between the explanatory variables and ALRI, crude and adjusted prevalence ratios (PRs) with 95% confidence intervals (CIs) were calculated using Poisson regression models with robust variance. ALRI was specified as the dependent variable. Crude PRs were estimated using separate bivariate robust Poisson regression models.

Variables with *p* < 0.20 in the bivariate analysis were selected for inclusion in the multivariable model. The adjusted model included child age, exclusive breastfeeding, birth-weight category, gestational age, complete immunization status, household overcrowding, maternal age, and passive smoking exposure. Adjusted PRs with 95% CIs were reported to quantify the independent association between each selected variable and ALRI.

All analyses were performed using Stata version 19.5. Statistical significance was set at *p* < 0.05.

### 2.7. Ethical Considerations

The study was conducted in accordance with the ethical principles of the Declaration of Helsinki and applicable Peruvian regulations for health research. The protocol was reviewed by the Institutional Committee of Ethics in Research of Universidad Privada San Juan Bautista, which issued approval/exemption under Constancia No. 2462-2024-CIEI-UPSJB. The project was classified as exempt from full protocol review because it did not qualify as research involving direct interaction with human subjects.

Institutional authorization was also granted by Hospital de Emergencias José Casimiro Ulloa through Oficio No. 000088-2025-HEJCU/DG, following review by the Institutional Committee of Ethics in Research of the hospital under Informe No. 004-2025-CIEI-HEJCU. The approved protocol was registered with code PI-034-2024 and titled “Factores asociados a infecciones respiratorias agudas en menores de 5 años en un hospital de emergencias de Lima, 2024.”

Because this was a retrospective study based exclusively on medical records, with no direct contact with patients or caregivers, informed consent was not required by the ethics review process. Confidentiality was maintained throughout the study. Each record was assigned a coded identifier, and no directly identifying personal information was included in the analytical database. Access to the database was restricted to the research team, and the results were reported only in aggregate form to prevent individual identification.

### 2.8. Use of Generative AI

No generative artificial intelligence tools were used to design the study, collect data, perform statistical analyses, or interpret the results. If language-assistance tools were used after manuscript drafting, their use was limited to grammar, clarity, and style editing and did not generate scientific content. The authors reviewed and approved the final manuscript and take full responsibility for its accuracy, integrity, and content.

## 3. Results

**Figure 1:**
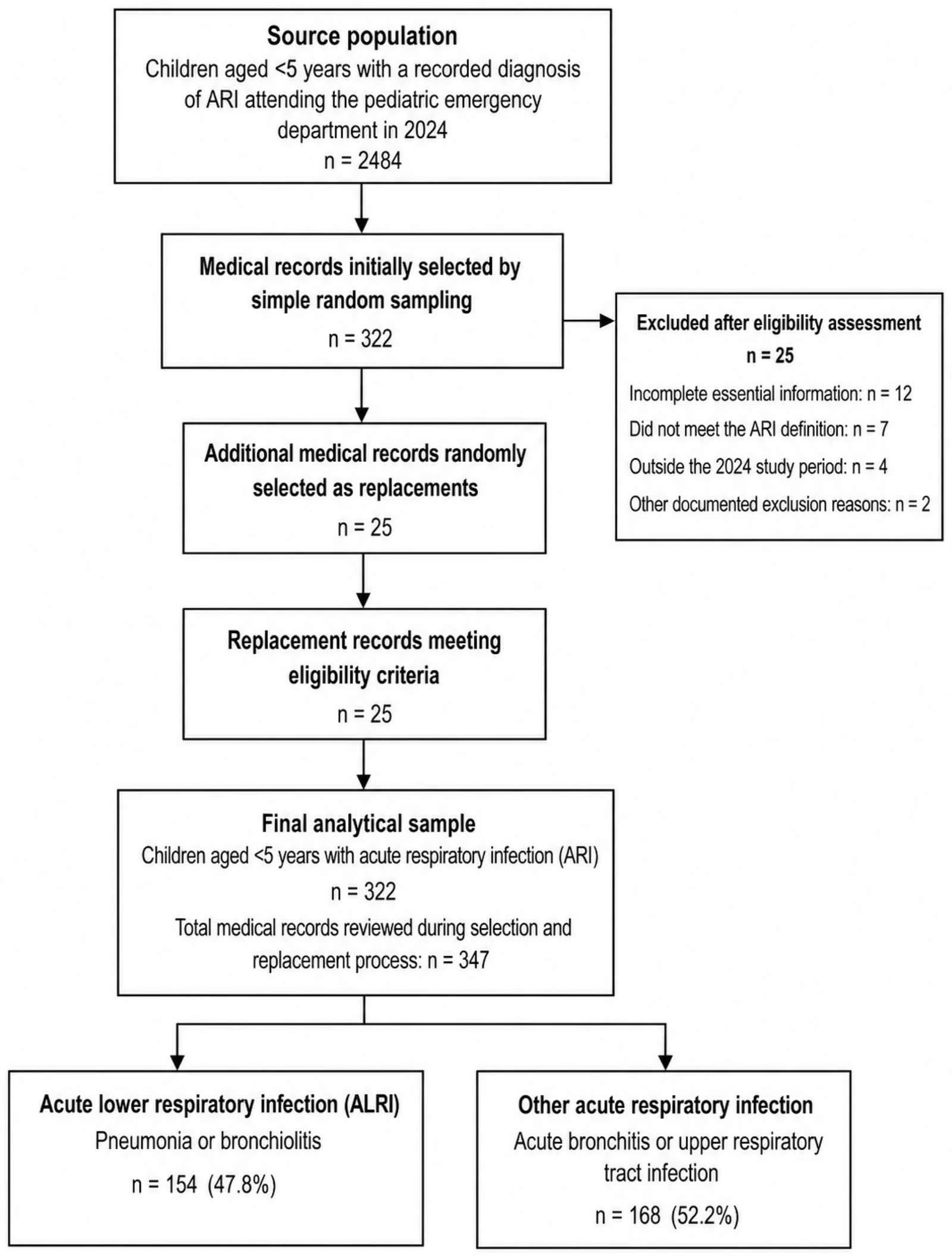
Flow diagram of medical record selection, eligibility assessment, replacement, and final analytical sample.

From a source population of 2484 children younger than five years with a recorded diagnosis of ARI who attended the pediatric emergency department during 2024, 322 medical records were initially selected by simple random sampling. During eligibility assessment, 25 records were excluded: 12 because of incomplete essential information, seven because they did not meet the study definition of acute respiratory infection, four because they did not correspond to the 2024 study period, and two for other documented exclusion reasons. Twenty-five additional records were then randomly selected as replacements, all of which met the eligibility criteria. Overall, 347 medical records were reviewed during the selection and replacement process, resulting in a final analytical sample of 322 children (Figure 1).

A total of 322 children under five years of age were included in the analysis. Most participants were aged 0 to <2 years (65.8%), and 71.4% were male. Exclusive breastfeeding was reported in 49.5% of children, while 52.8% had a history of preterm birth and 47.8% had incomplete immunizations. Regarding household and environmental characteristics, 53.4% of children lived in overcrowded households, 26.1% lived in prefabricated housing, and 24.0% had passive smoking exposure. Overall, ALRI was identified in 154 children, representing 47.8% of the study population (Table 1).

In the bivariate analysis, ALRI was more frequent among children aged 0 to <2 years than among those aged 2 to <5 years (72.7% vs. 27.3%; *p* = 0.017). Children without exclusive breastfeeding also had a higher frequency of ALRI than those who received exclusive breastfeeding (58.2% vs. 41.8%; *p* = 0.012). Significant differences were also observed according to birth weight (*p* = 0.023), gestational age (*p* = 0.012), immunization status (*p* = 0.004), and passive smoking exposure (*p* = 0.001). Household overcrowding (*p* = 0.163) and maternal age (*p* = 0.081) met the prespecified selection criterion for multivariable analysis, whereas sex, housing material, and maternal occupation did not (Table 2).

In the crude regression analysis, children aged 0 to <2 years had a higher prevalence of ALRI than those aged 2 to <5 years (PR = 1.38; 95% CI: 1.06–1.81). Similarly, absence of exclusive breastfeeding, extremely low birth weight, preterm birth, incomplete immunizations, and passive smoking exposure were associated with a higher prevalence of ALRI.

After adjustment in the multivariable robust Poisson regression model, children aged 0 to <2 years had a higher prevalence of ALRI (adjusted PR [aPR] = 1.27; 95% CI: 1.03–1.56). Absence of exclusive breastfeeding was also independently associated with a higher prevalence of ALRI (aPR = 1.24; 95% CI: 1.02–1.51). Compared with children with birth weight from 1500 to <2500 g, those with birth weight < 1000 g had a higher adjusted prevalence of ALRI (aPR = 1.43; 95% CI: 1.09–1.87). Incomplete immunizations were associated with a higher prevalence of ALRI (aPR = 1.28; 95% CI: 1.07–1.53), as was passive smoking exposure (aPR = 1.38; 95% CI: 1.15–1.65). In contrast, preterm birth, birth weight from 1000 to <1500 g, household overcrowding, and maternal age were not independently associated with ALRI after adjustment (Table 3).

**Figure 2:**
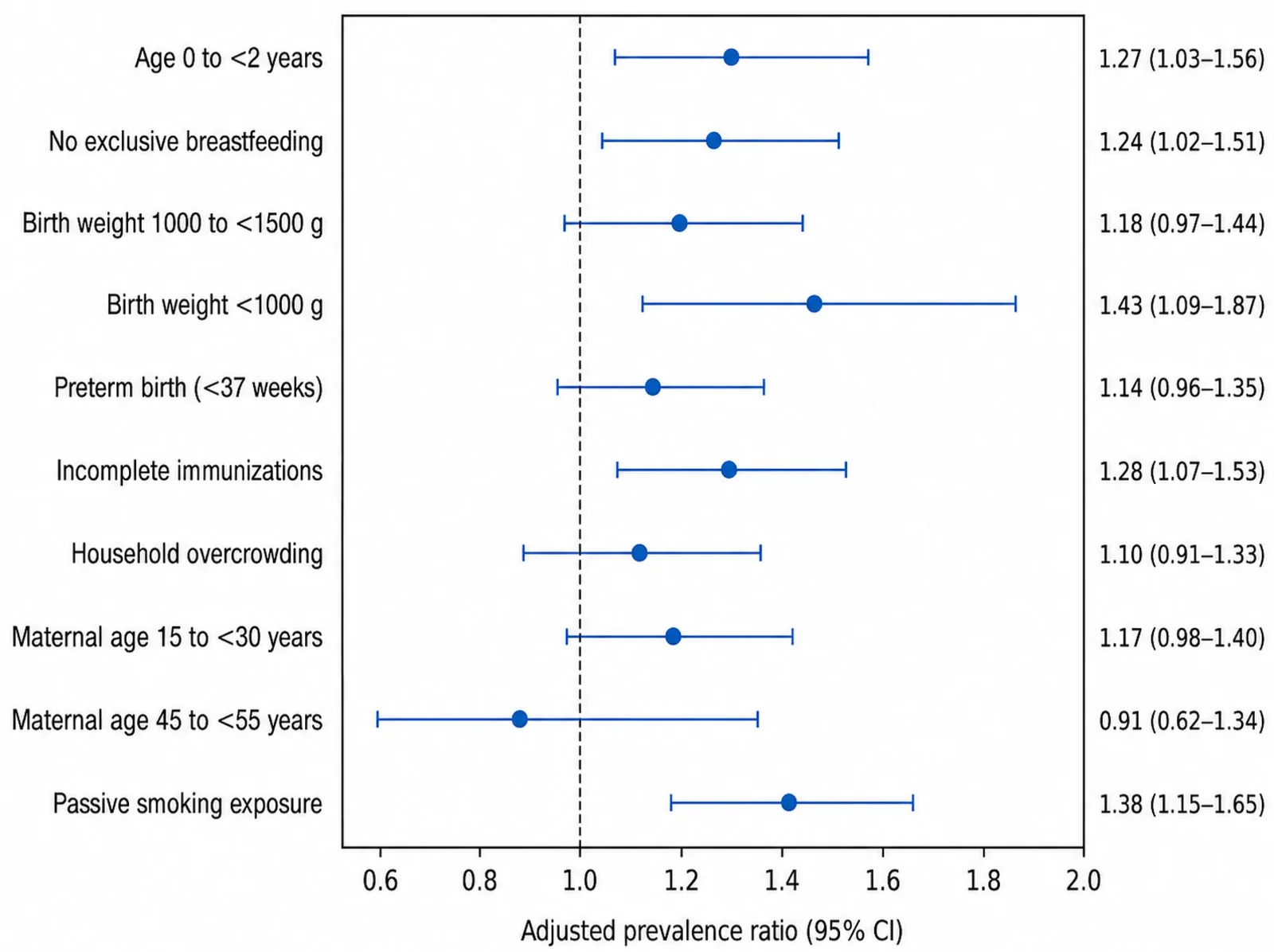
Adjusted prevalence ratios for acute lower respiratory infection among children under five years of age. Estimates were obtained from a multivariable robust Poisson regression model including variables with *p* < 0.20 in the bivariate analysis. Horizontal lines represent 95% confidence intervals.

Figure 2 summarizes the adjusted prevalence ratios for ALRI obtained from the multivariable model. The strongest adjusted associations were observed for birth weight < 1000 g, passive smoking exposure, incomplete immunizations, age 0 to <2 years, and absence of exclusive breastfeeding. The confidence intervals for preterm birth, household overcrowding, maternal age, and birth weight from 1000 to <1500 g crossed the null value, indicating no statistically significant association after adjustment (Figure 2).

## 4. Discussion

The present study found that ALRI accounted for nearly half of the acute respiratory infections evaluated among children under five years of age in this Peruvian emergency hospital. After adjustment for relevant perinatal, sociodemographic, and environmental factors, younger age, absence of exclusive breastfeeding, birth weight < 1000 g, incomplete immunizations, and passive smoking exposure remained independently associated with a higher prevalence of ALRI. These findings suggest that ALRI in this emergency-care population is associated with a combination of biological vulnerability, reduced early-life protection, incomplete preventive care, and household environmental exposure. In contrast, the associations observed for preterm birth, birth weight from 1000 to <1500 g, household overcrowding, and maternal age were attenuated after adjustment, suggesting that some crude associations may overlap with other perinatal and environmental characteristics. Overall, these results may help identify children with ARI who are more likely to present with lower respiratory tract involvement in emergency-care settings.

ALRI represented 47.8% of the ARI diagnoses included in our analytical sample. This proportion should be interpreted within the context of an emergency-hospital population and should not be directly compared with community-based estimates of ARI prevalence because the underlying populations, denominators, and case definitions differ. For example, Polo-Pucho et al. [14] evaluated caregiver-reported ARI symptoms in a nationally representative Peruvian survey, whereas Santri et al. [19] evaluated ARI symptoms using population-based survey data from Indonesia. We also reviewed the case definitions used in the hospital-based SARI studies cited for comparison. Aneja et al. [4] applied the WHO SARI definition, requiring hospitalization for an acute respiratory illness with fever or a history of fever and cough within a defined symptom-onset period, whereas Malhotra et al. [20] defined SARI as an acute respiratory illness of recent onset with fever, cough, and shortness of breath or difficulty breathing requiring hospitalization. These SARI definitions incorporate symptom-based and hospitalization criteria that were not used in our ALRI classification. Therefore, the findings of these studies provide relevant hospital-based epidemiological and etiological context but should not be considered direct comparators for the 47.8% proportion observed in our cohort. Our findings instead indicate that physician-recorded pneumonia and bronchiolitis constituted a substantial proportion of ARI diagnoses among children evaluated in this emergency-care setting.

Population-based studies from Peru and Indonesia have linked childhood ARI symptoms to household, nutritional, and sociodemographic conditions [14,21], whereas hospital-based surveillance from Colombia, Suriname, India, and Europe has shown substantial variation in respiratory pathogen profiles, case definitions, and clinical severity [22,23,24,25]. These differences further emphasize the importance of interpreting pediatric respiratory findings within the specific case definition, population, and healthcare setting of each study.

Children aged 0 to <2 years had a higher adjusted prevalence of ALRI in our study. This finding is consistent with evidence showing that lower respiratory infections requiring hospital care are concentrated early in life. In a Canadian cohort of 709 children hospitalized with RSV-associated ARI, Kirolos et al. [26] reported that 63.8% were younger than 2 years and that severe disease occurred more frequently at younger ages. Similarly, Li et al. [2] estimated 3.6 million RSV-associated ALRI hospital admissions globally among children aged 0–60 months in 2019, with the greatest mortality burden occurring during early infancy. Although Polo-Pucho et al. [14] found a different age pattern for caregiver-reported ARI symptoms in a nationally representative Peruvian survey, this difference may reflect variations in clinical setting, outcome definition, and disease spectrum. Younger children have greater biological vulnerability to lower respiratory infections, which provides a plausible context for the association observed in our study.

The absence of exclusive breastfeeding was independently associated with a higher prevalence of ALRI. This finding is consistent with previous evidence supporting a protective association between breastfeeding and childhood respiratory infections. In Bangladesh, exclusive breastfeeding was independently associated with lower odds of ARI among children under five years of age (AOR = 0.48; 95% CI: 0.28–0.82) [12]. More recently, an umbrella review including 12 systematic reviews and meta-analyses, 270 primary studies, and more than 10 million participants found that non-exclusive breastfeeding was associated with higher odds of pneumonia among children under five (OR = 2.34; 95% CI: 1.89–2.78) [27]. Sutriana et al. [28] similarly reported an association between non-exclusive breastfeeding and childhood pneumonia in Indonesia. These findings are consistent with the direction of our results and reinforce the importance of breastfeeding as an early-life preventive factor against clinically relevant respiratory infections.

Birth weight < 1000 g was independently associated with a higher prevalence of ALRI. Although previous studies have more commonly evaluated low birth weight or prematurity rather than the specific < 1000 g category, their findings support greater respiratory vulnerability among these children. Sutriana et al. [28] reported an association between low birth weight and childhood pneumonia in bivariate analysis, although the adjusted estimate was less precise. Among children hospitalized with RSV-associated respiratory infection, Kirolos et al. [26] found that prematurity was associated with severe disease among children younger than 2 years. Similarly, Wang et al. [17] documented a substantial burden of RSV-associated ALRI among preterm infants and young children. Our findings are consistent with this evidence and identify an association specifically among children with birth weight < 1000 g. However, this subgroup was relatively small, and the estimate should therefore be interpreted with appropriate caution.

This association should also be interpreted in light of unmeasured pediatric comorbidity. Bronchopulmonary dysplasia and other chronic lung conditions were not systematically collected as study variables. Although additional clinical history was documented in some medical records of children with birth weight < 1000 g, these data were incomplete and not uniformly available across the subgroup. Consequently, we could not determine the extent to which the observed association with extremely low birth weight may have been influenced by underlying prematurity-related respiratory morbidity.

Incomplete immunizations were independently associated with a higher prevalence of ALRI. This finding is consistent with previous studies linking incomplete childhood vaccination with respiratory morbidity. Islam et al. [12] found higher odds of ARI among children who were not immunized according to age than among fully immunized children, while Sutriana et al. [28] reported that incomplete basic immunization was associated with higher odds of childhood pneumonia. These findings support the direction of our results and reinforce the importance of maintaining age-appropriate immunization schedules as part of strategies to reduce preventable respiratory morbidity among young children.

Passive smoking exposure was the principal environmental factor independently associated with ALRI in our study. This finding is consistent with previous evidence linking household tobacco smoke exposure with childhood respiratory infections. In a hospital-based matched case–control study from Bangladesh, in-house smoking was independently associated with ARI among children under five [12]. Santri et al. [19] also reported an association between paternal smoking frequency and ARI symptoms among young children in Indonesia. In addition, the systematic review and meta-analysis by Jones et al. [29] found increased risk of lower respiratory infections associated with parental and household smoking exposure, while DiFranza et al. [30] reported an association between environmental tobacco smoke exposure and serious RSV-related lower respiratory tract disease. Together, these findings are consistent with the higher ALRI prevalence observed among smoke-exposed children in our study and support household smoke reduction as an important preventive measure. It should be noted that the passive smoking variable in our study reflected documented postnatal household tobacco smoke exposure and should not be interpreted as maternal smoking during pregnancy, which was not systematically recorded.

Preterm birth, household overcrowding, maternal age, and birth weight from 1000 to <1500 g were not independently associated with ALRI after adjustment. These findings do not necessarily contradict previous studies because differences in study populations, outcome definitions, and covariate adjustment may influence the observed associations. Islam et al. [12], for example, reported independent associations of preterm birth and household overcrowding with childhood ARI, whereas Polo-Pucho et al. [14] found that several sociodemographic and perinatal characteristics were not independently associated with caregiver-reported ARI symptoms in a Peruvian population-based analysis. Similarly, Sutriana et al. [28] observed an association between low birth weight and pneumonia in crude analysis that was attenuated after multivariable adjustment. In our study, the attenuation of these associations after adjustment suggests that they may overlap with other correlated perinatal, preventive, or environmental characteristics. Accordingly, absence of statistical significance should not be interpreted as evidence that these factors lack clinical relevance.

The contribution of this study lies primarily in its clinical and geographic context rather than in identifying previously unrecognized risk factors for childhood respiratory infection. Breastfeeding, immunization, low birth weight or prematurity, and exposure to tobacco smoke are established determinants of pediatric respiratory health. However, the magnitude and independence of their associations may vary across populations and healthcare settings. In this Peruvian emergency-hospital population, simultaneous multivariable assessment showed that younger age, absence of exclusive breastfeeding, birth weight < 1000 g, incomplete immunizations, and passive smoking exposure remained independently associated with ALRI, whereas preterm birth and household overcrowding did not retain independent associations after adjustment. These findings provide context-specific estimates for a population that is not well represented by community-based surveys and may help inform risk assessment and preventive counseling in comparable emergency-care settings.

The main strengths of this study include its focus on children under five years of age, the evaluation of ALRI using physician-recorded diagnoses, and the simultaneous assessment of perinatal, breastfeeding, immunization, sociodemographic, and environmental factors within the same analytical framework. The hospital-based design also provides evidence from a real-world pediatric emergency setting in Peru, where information on factors associated with lower respiratory infections remains limited.

Several limitations should be acknowledged. First, the cross-sectional design precludes causal inference and allows only associations with ALRI prevalence to be evaluated. Second, because the study was conducted at a single emergency hospital, the findings may not be generalizable to community populations or other healthcare settings. Third, outcome classification was based on physician-recorded final diagnoses obtained retrospectively from medical records rather than on prospective diagnostic adjudication using standardized research criteria. Although imaging modalities such as chest radiography and lung ultrasound may contribute to the diagnostic evaluation of pediatric pneumonia, recent evidence supports the potential role of lung ultrasound in both diagnosis and monitoring [31]. Information on the imaging modality used, however, was not systematically collected in our study. Therefore, we could not determine whether individual pneumonia diagnoses were supported by chest radiography, lung ultrasound, or clinical assessment alone, and some diagnostic misclassification cannot be excluded. Similarly, variables obtained from medical records or caregiver-reported information may be subject to information bias.

Fourth, residual confounding remains possible because socioeconomic, nutritional, environmental, healthcare-access, and pediatric comorbidity characteristics were not comprehensively captured in the available records. Pediatric comorbidities were not systematically collected as study variables, and bronchopulmonary dysplasia or other chronic lung disease was not consistently documented for analytical purposes. Although some medical records, including those of children with birth weight < 1000 g, contained additional clinical history, this information was incomplete and not uniformly available across participants. Therefore, the potential confounding effect of chronic underlying conditions could not be evaluated, which is particularly relevant when interpreting associations involving prematurity and extremely low birth weight.

In addition, pathogen-specific etiological data were not systematically available; therefore, we could not evaluate whether the observed associations differed according to viral or bacterial etiology. Maternal smoking during pregnancy was also not systematically recorded, and the passive smoking variable represented postnatal household exposure rather than prenatal tobacco exposure. These unmeasured factors may have contributed to residual confounding and clinical heterogeneity within the ALRI group. Although the final sample exceeded the complementary analytical sample-size estimate, limited statistical power for detecting smaller associations or effects within infrequent exposure categories cannot be excluded. Finally, variables that were not statistically significant after adjustment may remain clinically relevant, particularly when their effects overlap with other correlated perinatal or environmental factors included in the model.

## 5. Conclusions

In this study, ALRI accounted for nearly half of the acute respiratory infections evaluated among children under five years of age in a Peruvian emergency hospital. Age 0 to <2 years, absence of exclusive breastfeeding, birth weight < 1000 g, incomplete immunizations, and passive smoking exposure were independently associated with a higher prevalence of ALRI. These findings highlight the importance of early-life preventive measures, including breastfeeding promotion, timely immunization, follow-up of extremely low-birth-weight children, and reduction in household tobacco smoke exposure. In emergency-care settings, these characteristics may help identify children with acute respiratory illness who are more likely to present with lower respiratory tract involvement and may benefit from closer clinical assessment and preventive counseling.

## Figures and Tables

**Table 1:** Baseline characteristics of the study population.

Variable	*n* (%)
**Host and perinatal factors**	
Child age	
0 to <2 years	212 (65.8)
2 to <5 years	110 (34.2)
Sex	
Male	230 (71.4)
Female	92 (28.6)
Exclusive breastfeeding	
Yes	159 (49.5)
No	162 (50.5)
Low birth weight	
1500 to <2500 g	136 (42.4)
1000 to <1500 g	160 (49.8)
<1000 g	25 (7.8)
Gestational age	
Term, ≥37 weeks	152 (47.2)
Preterm, <37 weeks	170 (52.8)
Complete immunizations	
Yes	168 (52.2)
No	154 (47.8)
**Sociodemographic factors**	
Household overcrowding	
Yes	172 (53.4)
No	150 (46.6)
Housing material	
Masonry/permanent material	238 (73.9)
Prefabricated material	84 (26.1)
Maternal age	
15 to <30 years	137 (42.5)
30 to <45 years	143 (44.4)
45 to <55 years	42 (13.0)
Maternal occupation	
Homemaker	78 (24.2)
Part-time work	172 (53.4)
Full-time work	72 (22.4)
**Environmental factors**	
Passive smoking exposure	
Yes	77 (24.0)
No	244 (76.0)
Acute lower respiratory infection (ALRI)	
Yes	154 (47.8%)
No	168 (52.2%)

Note. Percentages were calculated using available non-missing data. Missing values were <1% for exclusive breastfeeding, birth weight, passive smoking exposure, and district of origin. ALRI, acute lower respiratory infection.

**Table 2:** Bivariate associations between acute lower respiratory infection and explanatory variables.

Variable	Other ARI, *n* (%)	ALRI, *n* (%)	*p*-Value
**Host and perinatal factors**			
Child age			0.017
0 to <2 years	100 (59.5)	112 (72.7)	
2 to <5 years	68 (40.5)	42 (27.3)	
Sex			0.902
Male	119 (70.8)	111 (72.1)	
Female	49 (29.2)	43 (27.9)	
Exclusive breastfeeding			0.012
Yes	95 (56.5)	64 (41.8)	
No	73 (43.5)	89 (58.2)	
Low birth weight			0.023
1500 to <2500 g	81 (48.2)	55 (35.9)	
1000 to <1500 g	79 (47.0)	81 (52.9)	
<1000 g	8 (4.8)	17 (11.1)	
Gestational age			0.012
Term, ≥37 weeks	91 (54.2)	61 (39.6)	
Preterm, <37 weeks	77 (45.8)	93 (60.4)	
Complete immunizations			0.004
Yes	101 (60.1)	67 (43.5)	
No	67 (39.9)	87 (56.5)	
**Sociodemographic factors**			
Household overcrowding			0.163
No	85 (50.6)	65 (42.2)	
Yes	83 (49.4)	89 (57.8)	
Housing material			0.736
Masonry/permanent material	126 (75.0)	112 (72.7)	
Prefabricated material	42 (25.0)	42 (27.3)	
Maternal age			0.081
15 to <30 years	62 (36.9)	75 (48.7)	
30 to <45 years	80 (47.6)	63 (40.9)	
45 to <55 years	26 (15.5)	16 (10.4)	
Maternal occupation			0.586
Homemaker	37 (22.0)	41 (26.6)	
Part-time work	91 (54.2)	81 (52.6)	
Full-time work	40 (23.8)	32 (20.8)	
**Environmental factors**			
Passive smoking exposure			0.001
No	141 (83.9)	103 (67.3)	
Yes	27 (16.1)	50 (32.7)	

Note. ALRI was defined as a physician-recorded final diagnosis of pneumonia or bronchiolitis. Other ARI comprised acute bronchitis or upper respiratory tract infection. Percentages are column percentages. *p*-values were calculated using Pearson’s chi-square test or Fisher’s exact test, as appropriate. Variables with *p* < 0.20 in the bivariate analysis were selected for the multivariable model. ALRI, acute lower respiratory infection; ARI, acute respiratory infection.

**Table 3:** Crude and adjusted prevalence ratios for acute lower respiratory infection.

Variable	Crude PR (95% CI)	*p*-Value	Adjusted PR (95% CI)	*p*-Value
Child age				
2 to <5 years	Reference		Reference	
0 to <2 years	1.38 (1.06–1.81)	0.018	1.27 (1.03–1.56)	0.026
Exclusive breastfeeding				
Yes	Reference		Reference	
No	1.36 (1.08–1.73)	0.010	1.24 (1.02–1.51)	0.031
Low birth weight				
1500 to <2500 g	Reference		Reference	
1000 to <1500 g	1.25 (0.97–1.62)	0.084	1.18 (0.97–1.44)	0.096
<1000 g	1.68 (1.20–2.36)	0.003	1.43 (1.09–1.87)	0.010
Gestational age				
Term, ≥37 weeks	Reference		Reference	
Preterm, <37 weeks	1.36 (1.07–1.73)	0.011	1.14 (0.96–1.35)	0.129
Complete immunizations				
Yes	Reference		Reference	
No	1.42 (1.12–1.79)	0.003	1.28 (1.07–1.53)	0.007
Household overcrowding				
No	Reference		Reference	
Yes	1.19 (0.95–1.51)	0.136	1.10 (0.91–1.33)	0.322
Maternal age				
30 to <45 years	Reference		Reference	
15 to <30 years	1.24 (0.98–1.58)	0.075	1.17 (0.98–1.40)	0.084
45 to <55 years	0.86 (0.56–1.33)	0.505	0.91 (0.62–1.34)	0.631
Passive smoking exposure				
No	Reference		Reference	
Yes	1.54 (1.23–1.92)	<0.001	1.38 (1.15–1.65)	0.001

Note. PR, prevalence ratio; aPR, adjusted prevalence ratio; CI, confidence interval; ALRI, acute lower respiratory infection. Crude estimates were obtained from bivariate robust Poisson regression models. Adjusted estimates were obtained from a multivariable robust Poisson regression model. Only variables with *p* < 0.20 in the bivariate analysis were included in the multivariable model: child age, exclusive breastfeeding, birth weight, gestational age, complete immunizations, household overcrowding, maternal age, and passive smoking exposure.

## Data Availability

The data used in this study were obtained from retrospective review of medical records from Hospital de Emergencias José Casimiro Ulloa and are not publicly available due to confidentiality, privacy, and ethical restrictions. The de-identified dataset and analytic syntax may be made available from the corresponding author upon reasonable request, subject to institutional authorization and applicable ethical regulations.

## Abbreviations

The following abbreviations are used in this manuscript:

ALRI	Acute lower respiratory infection
ARI	Acute respiratory infection
CIEI-HEJCU	Institutional Research Ethics Committee of Hospital de Emergencias José Casimiro Ulloa
CIEI-UPSJB	Institutional Research Ethics Committee of Universidad Privada San Juan Bautista
RSV	Respiratory syncytial virus
SARI	Severe acute respiratory infection

## References

[1] Perin J., Mulick A., Yeung D., Villavicencio F., Lopez G., Strong K.L., Prieto-Merino D., Cousens S., Black R.E., Liu L. (2022). Global, Regional, and National Causes of under-5 Mortality in 2000-19: An Updated Systematic Analysis with Implications for the Sustainable Development Goals. Lancet Child Adolesc. Health.

[2] Li Y., Wang X., Blau D.M., Caballero M.T., Feikin D.R., Gill C.J., Madhi S.A., Omer S.B., Simões E.A.F., Campbell H. (2022). Global, Regional, and National Disease Burden Estimates of Acute Lower Respiratory Infections Due to Respiratory Syncytial Virus in Children Younger than 5 Years in 2019: A Systematic Analysis. Lancet.

[3] Sini de Almeida R., Leite J., Atwell J.E., Elsobky M., LaRotta J., Mousa M., Thakkar K., Fletcher M.A. (2024). Respiratory Syncytial Virus Burden in Children under 2 Years Old in Understudied Areas Worldwide: Gap Analysis of Available Evidence, 2012–2022. Front. Pediatr..

[4] Aneja S., Singh V., Narayan V.V., Gohain M., Choudekar A., Gaur B., DeBord K.R., Whitaker B., Krishnan A., Broor S. (2024). Respiratory Viruses Associated with Severe Acute Respiratory Infection in Children Aged <5 Years at a Tertiary Care Hospital in Delhi, India during 2013–15. J. Glob. Health.

[5] Ramírez-Soto M.C., Ortega-Cáceres G., Garay-Uribe J. (2022). Characteristics of Respiratory Syncytial Virus versus Influenza Infection in Hospitalized Patients of Peru: A Retrospective Observational Study. Trop. Med. Infect. Dis..

[6] del Valle Mendoza J., Cornejo-Tapia A., Weilg P., Verne E., Nazario-Fuertes R., Ugarte C., del Valle L.J., Pumarola T. (2015). Incidence of Respiratory Viruses in Peruvian Children with Acute Respiratory Infections. J. Med. Virol..

[7] Pavic-Espinoza I., Bendezú-Medina S., Herrera-Alzamora A., Weilg P., Pons M.J., Aguilar-Luis M.A., Petrozzi-Helasvuo V., del Valle Mendoza J. (2015). High Prevalence of Bordetella Pertussis in Children under 5 Years Old Hospitalized with Acute Respiratory Infections in Lima, Peru. BMC Infect. Dis..

[8] Kandeel A., Fahim M., Deghedy O., Roshdy W.H., Khalifa M.K., El Shesheny R., Kandeil A., Wagdy S., Naguib A., Afifi S. (2023). Multicenter Study to Describe Viral Etiologies, Clinical Profiles, and Outcomes of Hospitalized Children with Severe Acute Respiratory Infections, Egypt 2022. Sci. Rep..

[9] Instituto Nacional de Estadística e Informática (INEI) (2025). Encuesta Demográfica y de Salud Familiar 2024: Departamento de Lima.

[10] Deng S., Cong B., Edgoose M., De Wit F., Nair H., Li Y. (2024). Risk Factors for Respiratory Syncytial Virus-Associated Acute Lower Respiratory Infection in Children under 5 Years: An Updated Systematic Review and Meta-Analysis. Int. J. Infect. Dis..

[11] Desye B., Geto A.K., Daba C., Berihun G., Berhanu L. (2025). Indoor Air Pollution Exposure and Acute Respiratory Infection among Under-Five Children in Low- and Middle-Income Countries: A Systematic Review and Meta-Analysis of Epidemiological Studies. BMC Infect. Dis..

[12] Islam M., Islam K., Dalal K., Hossain Hawlader M.D. (2024). In-House Environmental Factors and Childhood Acute Respiratory Infections in under-Five Children: A Hospital-Based Matched Case-Control Study in Bangladesh. BMC Pediatr..

[13] Nshimiyimana Y., Zhou Y. (2022). Analysis of Risk Factors Associated with Acute Respiratory Infections among Under-Five Children in Uganda. BMC Public Health.

[14] Polo-Pucho D.A., Gonzales-Carrillo J.J., Arce-Huamani M.A. (2025). Factors Associated with Acute Respiratory Infections in Children Under Five Years Old: Analysis of the Demographic and Family Health Survey. Children.

[15] Ministerio de Salud Cada Noche se Atienden Más de 100 Casos de Emergencias y Urgencias en el Hospital Casimiro Ulloa. https://www.gob.pe/institucion/minsa/noticias/741264-cada-noche-se-atienden-mas-de-100-casos-de-emergencias-y-urgencias-en-el-hospital-casimiro-ulloa.

[16] Bhurtel R., Pokhrel R.P., Kalakheti B. (2022). Acute Respiratory Infections among Under-Five Children Admitted in a Tertiary Hospital of Nepal: A Descriptive Cross-Sectional Study. JNMA J. Nepal Med. Assoc..

[17] Wang X., Li Y., Shi T., Bont L.J., Chu H.Y., Zar H.J., Wahi-Singh B., Ma Y., Cong B., Sharland E. (2024). Global Disease Burden of and Risk Factors for Acute Lower Respiratory Infections Caused by Respiratory Syncytial Virus in Preterm Infants and Young Children in 2019: A Systematic Review and Meta-Analysis of Aggregated and Individual Participant Data. Lancet.

[18] Instituto Nacional de Estadística e Informática (INEI) Fichas Técnicas de Los Indicadores Generados a Partir de La Encuesta Nacional de Hogares—ENAHO: Población En Viviendas Con Hacinamiento. https://www.gob.pe/institucion/inei/informes-publicaciones/4833930-pobreza-multidimensional-revision-2023.

[19] Santri I.N., Wardani Y., Phiri Y.V.A., Nyam G., Putri T.A., Isni K., Suryani D., Sambo G. (2023). Associations Between Indoor Air Pollutants and Risk Factors for Acute Respiratory Infection Symptoms in Children Under 5: An Analysis of Data from the Indonesia Demographic Health Survey. J. Prev. Med. Public Health.

[20] Malhotra B., Swamy M.A., Janardhan Reddy P.V., Gupta M.L. (2016). Viruses Causing Severe Acute Respiratory Infections (SARI) in Children ≤5 Years of Age at a Tertiary Care Hospital in Rajasthan, India. Indian J. Med. Res..

[21] Purnama T.B., Wagatsuma K., Saito R. (2025). Prevalence and Risk Factors of Acute Respiratory Infection and Diarrhea among Children under 5 Years Old in Low-Middle Wealth Household, Indonesia. Infect. Dis. Poverty.

[22] Iles-Dorado E., Alarcón Soto J., Aguirre J.L., Trochez A.I., Trochez M.J., Ordoñez-Betancourth J.E., Moreno M., Romero-Fernández S., Pérez-Camacho P.M., Orrego Flórez M.S. (2025). Sentinel Surveillance of Acute Respiratory Infection (ARI) in Children under Five Years Old from 2015 to 2022 at a High-Complexity Health Care Institution in Cali, Colombia. J. Glob. Health.

[23] Juliana A.E., Tang M.-J., Kemps L., Noort A.C., Hermelijn S., Plötz F.B., Zonneveld R., Wilschut J.C. (2021). Viral Causes of Severe Acute Respiratory Infection in Hospitalized Children and Association with Outcomes: A Two-Year Prospective Surveillance Study in Suriname. PLoS ONE.

[24] Deval H., Srivastava M., Srivastava N., Kumar N., Agarwal A., Potdar V., Mehta A., Sharma B., Beniwal R., Singh R. (2024). Hospital-Based Surveillance of Respiratory Viruses Among Children Under Five Years of Age with ARI and SARI in Eastern UP, India. Viruses.

[25] Orosz N., Gömöri G., Battamir U., Nagy A.C. (2024). Hospital-Based Cross-Sectional Study on the Clinical Characteristics of Children with Severe Acute Respiratory Infections in Hungary. BMC Infect. Dis..

[26] Kirolos N., Mtaweh H., Datta R.R., Farrar D.S., Seaton C., Bone J.N., Muttalib F., Kaziev C.L., Fortini J., Mahant S. (2025). Risk Factors for Severe Disease Among Children Hospitalized with Respiratory Syncytial Virus. JAMA Netw. Open.

[27] Abate B.B., Tusa B.S., Sendekie A.K., Araya F.G., Bizuayehu M.A., Walle G.T., Kitaw T.A., Tilahun B.D., Alamaw A.W., Zemariam A.B. (2025). Non-Exclusive Breastfeeding Is Associated with Pneumonia and Asthma in under-Five Children: An Umbrella Review of Systematic Review and Meta-Analysis. Int. Breastfeed. J..

[28] Sutriana V.N., Sitaresmi M.N., Wahab A. (2021). Risk Factors for Childhood Pneumonia: A Case-Control Study in a High Prevalence Area in Indonesia. Clin. Exp. Pediatr..

[29] Jones L.L., Hashim A., McKeever T., Cook D.G., Britton J., Leonardi-Bee J. (2011). Parental and Household Smoking and the Increased Risk of Bronchitis, Bronchiolitis and Other Lower Respiratory Infections in Infancy: Systematic Review and Meta-Analysis. Respir. Res..

[30] DiFranza J.R., Masaquel A., Barrett A.M., Colosia A.D., Mahadevia P.J. (2012). Systematic Literature Review Assessing Tobacco Smoke Exposure as a Risk Factor for Serious Respiratory Syncytial Virus Disease among Infants and Young Children. BMC Pediatr..

[31] Isac R., Cugerian-Ratiu A.-M., Micsescu-Olah A.-M., Bodescu A.D., Vlad L.-A., Zaroniu A.M., Gafencu M., Doros G. (2025). Can Lung Ultrasound Act as a Diagnosis and Monitoring Tool in Children with Community Acquired Pneumonia? Correlation with Risk Factors, Clinical Indicators and Biologic Results. J. Clin. Med..

